# Redox/pH-Responsive Biodegradable Thiol-Hyaluronic Acid/Chitosan Charge-Reversal Nanocarriers for Triggered Drug Release

**DOI:** 10.3390/polym13213785

**Published:** 2021-10-31

**Authors:** Dandan Xia, Feilong Wang, Shuo Pan, Shenpo Yuan, Yunsong Liu, Yongxiang Xu

**Affiliations:** 1 Department of Dental Materials, Peking University School and Hospital of Stomatology, Beijing 100081, China; dandan66x@126.com (D.X.); yuanshenpo@163.com (S.Y.); 2 National Engineering Laboratory for Digital and Material Technology of Stomatology, National Clinical Research Center for Oral Diseases, Beijing Key Laboratory of Digital Stomatology, National Medical Products Administration Key Laboratory for Dental Materials, Research Center of Engineering and Technology for Digital Dentistry, Ministry of Health, Beijing 100081, China; drwangfeilong@126.com; 3 Department of Prosthodontics, Peking University School and Hospital of Stomatology, Beijing 100081, China; 4 Center for Medical Device Evaluation, National Medical Products Administration, Haidian District, Beijing 100081, China; panshuo@cmde.org.cn

**Keywords:** drug-release nanocarrier, dual-stimuli responsive, charge reversal, thiol-hyaluronic acid, chitosan

## Abstract

Biodegradable nanoparticles and micelles are promising nanosystems for the targeted delivery of potent anticancer drugs. By using specialized polymers as nanocarriers, targeted drug delivery and release can be developed. We developed thiol-hyaluronic acid (HA-SH)/chitosan (CS) nanoparticles with redox/pH dual-responsiveness via electrostatic self-assembly followed by spontaneous chemical cross-linking. The nanoparticle surface charges were reversible through different HA-SH and CS mass ratios. Doxorubicin (DOX) was used as a model drug. Dual cross-linked nanoparticles with diameters of approximately 300 nm exhibited superior stability under physiological conditions compared with nanoparticles without disulfide cross-linking. DOX was loaded more efficiently into negative nanoparticles (45.7 wt%) than positive nanoparticles (14.2 wt%). Drug release from negative nanoparticles (ζ potential of approximately −20) was higher (87.8 wt%) at pH 4.5 and in the presence of 10 mM glutathione. Positive nanoparticles (ζ potential of approximately +20) showed the same trend, but the release rate was slower than that of negative nanoparticles. DOX-loaded HA-SH/CS particles were taken up by human breast cancer cells (SKBR3), and the loaded drug was released, exhibiting potential antitumor efficacy. The HA-SH/CS nanoparticles in this study were stable under physiological conditions and are promising candidates for the targeted delivery and release of anticancer drugs.

## 1. Introduction

Recently, biodegradable nanoparticles and micelles have emerged as one of the most promising nanosystems for the controlled and targeted delivery of potent anticancer drugs. They allow for responding to specific microenvironmental changes of cancer cells and quick drug release, which are highly desired functions in cancer treatments with minimal undesired effects on normal cells [1,2,3,4,5,6]. These systems can be designed for a specific purpose and to control drug biodistribution in response to specific stimuli, either exogenous stimuli including variations in temperature, magnetic field, ultrasound intensity, light, or electric pulses, and endogenous stimuli, such as changes in pH, enzyme concentration, or redox gradients [7,8,9,10]. Natural and synthetic polymers are currently the most extensively explored materials for constructing nanoparticle-based drug carriers [11,12].

Hyaluronic acid (HA), or hyaluronan, is a linear polysaccharide that consists of alternating units of repeating disaccharide, β-1,4-D-glucuronic acid–β-1,3-N-acetyl-D-glucosamine. HA is found throughout the body from the vitreous of the eye to the extracellular matrix of cartilage tissues. The United States Food and Drug Administration (FDA) has approved the use of HA for certain eye surgeries. Its biodegradable, nontoxic, nonimmunogenic, and noninflammatory properties make HA an ideal carrier polymer for systemic drug delivery applications [13]. 

The HA-specific receptor CD44 and receptor for hyaluronan-mediated motility (RHAMM) are found at low levels on epithelial, hematopoietic, and neuronal cells, but they are overexpressed in almost all types of cancer cells [14,15]. The relationships between tumor cells and HA receptors make HA a potential ligand for the targeted therapy of tumors. Consequently, nanocarriers using HA as the primary targeting constituent have been widely investigated for drug delivery [16]. Coating nanoparticles, such as chitosan (CS) nanoparticles, liposome nanoparticles, and calcium phosphate nanoparticles, with HA, may also reduce cytotoxicity [17,18,19]. 

HA can be easily modified chemically because of its unique chemical structure. Thiol-HA (HA-SH) is synthesized through the modification of the carboxylate groups, with the thiol potentially being oxidized to form disulfide bonds [20]. Disulfide bonds are prone to rapid cleavage by reduced glutathione (GSH) and can be used to attain redox sensitivity. GSH concentrations differ extracellularly (2–10 μM) compared with that in intracellular compartments (2–10 mM), and tumor tissues compared with that in healthy tissues [21].

HA can exist as a polyanion and form polyelectrolyte complexes with positive polymers, such as CS, silk fibroin, and collagen. CS is the only natural positively charged polysaccharide. It is the second-most abundant polysaccharide in nature and has many biological properties, including immune enhancement, antimicrobial, and antitumor properties [22]. HA-CS polyelectrolyte complexes, including fibers, films, microparticles, and hydrogels, have been studied by many researchers for use in tissue engineering and drug release [23,24]. However, polyelectrolyte complexes are unstable and readily dissociate at a neutral pH and high ionic strength due to the reduced protonation of CS and disruption of polymer/polymer ionic contacts [25].

**Scheme 1:**
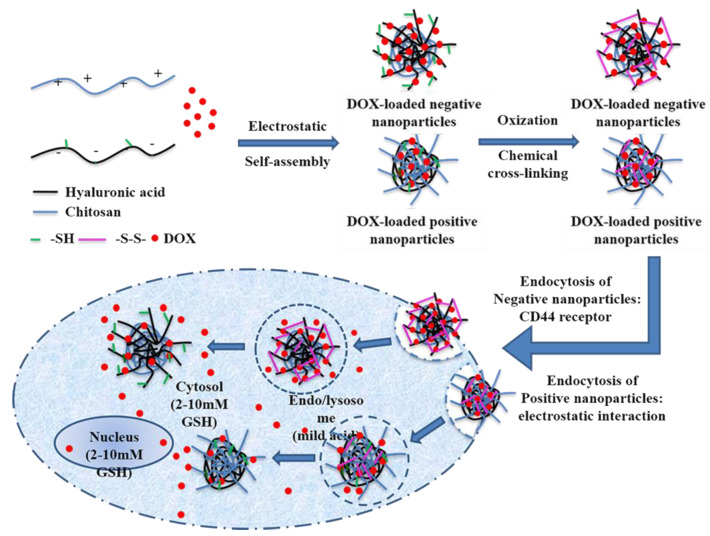
Schematic illustration of preparation, redox and pH-sensitive drug release of HA-SH-CS nanoparticles.

In our previous study, tissue scaffolds were prepared using CS. The structure and properties of the CS scaffolds were fabricated through a step-by-step gelling process and second-most [26]. The design of the HA-modified nanocarriers has been proven to be effective for targeting CD44-overexpressing tumor cells. Nanocarriers with unique physical properties and elaborate designs can improve the bioavailability and therapeutic efficacy of antitumor drugs. In the present study, biocompatible HA nanoparticles were prepared using CS as the physical cross-linker and disulfide as the chemical cross-linker and then used as targeted nanocarriers. Using doxorubicin (DOX) as a model drug, the redox/pH dual stimuli-responsive release by the HA-SH/CS nanoparticles was investigated (Scheme 1). It was hypothesized that DOX-loaded HA-SH/CS nanoparticles, with their polyelectrolyte complexes and disulfide cross-linking, would be stable under physiological conditions, preferentially accumulate at the target site, and release their payload in the acid-cleavable and glutathione-reducing microenvironments of cancer cells. 

## 2. Materials and Methods

### 2.1. Materials

HA (MW = 4–6 kDa) was purchased from Bloomage Freda Biopharm Co., Ltd. (Jinan, China). The 1-ethyl-3-(3-dimethylaminopropyl)-carbodiimide (EDC), cysteamine dihydrochloride, 1-hydroxybenzotriazole (HOBt), DL-1,4-Dithiothreitol (DTT), CS (low molecular weight), and GSH used in this study were purchased from Sigma-Aldrich (St. Louis, MO, USA). H_2_O_2_ was purchased from Fisher Scientific Company LLC (Pittsburgh, PA, USA). Doxorubicin hydrochloride was purchased from Melone Pharmaceutical Co., Ltd. (Dalian, China). Cell culture reagents and supplies, fetal bovine serum (FBS), and Hoechst 33,258 were purchased from Life Technologies (New York, NY, USA).

### 2.2. HA-SH Synthesis and Characterization 

HA-SH was synthesized and characterized as previously described [27]. HA (200 mg) was dissolved in 50 mL deionized water (0.4% *w/v* solution). EDC (520 mg) and HOBt (460 mg) were added to the solution and stirred for 1 h. Cysteamine dihydrochloride (600 mg) was added, and the pH was adjusted to 6.8 with 1.0 M NaOH. The reaction was then allowed to proceed overnight at 23 °C under constant stirring. The reaction products were poured into a pre-washed dialysis membrane tube (MW*_CO_* = 3.5 kDa) and dialyzed against an excess volume of water. DTT (600 mg) was then added to the dialyzed solution at pH 8.5 to reduce the disulfide bonds of the cysteamine-grafted HA. After stirring for 4 h, the pH of the solution was dropped to 3.5 by adding 0.1 M HCl. Finally, the HA-SH was precipitated in excess ethanol, re-dissolved in water, and freeze-dried for 3 d. The conjugated HA containing free thiol groups was obtained as a white foam (yield of 83%). The structures of the resulting products were identified by proton nuclear magnetic resonance (^1^H-NMR) in heavy water (deuterium oxide) using a Varian MR400 spectrometer (Agilent Technologies, Santa Clara, CA, USA). The degree of substitution, defined as the number of free thiol groups per 100 repeat units of HA, and the disulfide group content were determined using the Ellman method [28]. 

### 2.3. HA-SH/CS Nanoparticle Preparation 

**Scheme 2:**
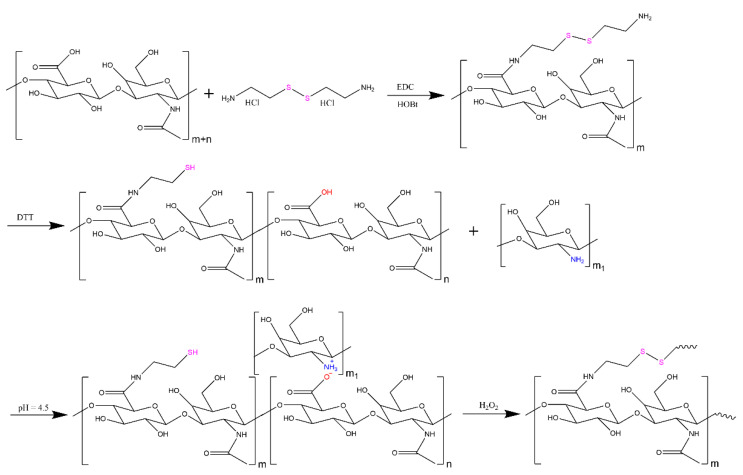
Synthesis and preparation steps for the preparation of HA-SH/CS nanoparticles.

Stimuli-responsive nanocarriers were prepared via a three-step process (Scheme 2). In the first step, cysteamine dihydrochloride, via its primary amine groups, was reacted with the carboxylic groups of hyaluronic acid with EDC/HOBt coupling protocol. DTT was used to reduce the disulfide bonds of the cysteamine-grafted HA in the second step. The third step consisted of electrostatic self-assembly and oxidation of the thiol groups.

Solutions of 0.5% (*w*/*v*) HA-SH were prepared directly in deionized water under magnetic stirring overnight. CS was dispersed in deionized water at a concentration of 0.5% (*w*/*v*) by adding stoichiometric amounts of acetic acid with respect to free amino groups under magnetic stirring overnight. Before use, all solutions were adjusted to pH = 4.5 with 0.1 M NaOH or HCl as necessary and filtered through 0.22-μm pore-size membranes. 

The HA-SH/CS nanoparticles were formed by a fast one-shot addition of the components and magnetic stirring (500 rpm) at 23 °C under different stoichiometric conditions. The initial mass mixing ratio (R) of CS and HA-SH (m_CS_/m_HA-SH_) ranged from 0.05 to 0.70. Three molar excesses of 3% H_2_O_2_ relative to the thiol group content were then added to form disulfide bonds. The solutions were stirred for 3 h and then allowed to sit undisturbed overnight. A subsequent evaluation of the nanoparticle physical stability was conducted, and particle loading with DOX was performed using two mass mixing ratios, R = 0.10 (negative nanoparticles) and R = 0.50 (positive nanoparticles).

### 2.4. HA-SH/CS Nanoparticle Characterization

The contents of free thiol and disulfide groups in the HA-SH/CS nanoparticles after oxidization were also evaluated using the Ellman method. The mean particle size, size distribution, and ζ potential values of the nanoparticles were determined using a Delsa^TM^ Nano C particle analyzer (Beckman Coulter, Inc. Carlsbad, CA, USA). The turbidity of the polyelectrolyte complex dispersions was characterized according to optical density measured at 500 nm (OD_500_) using UV/VIS spectrometer (Hitachi U-2910, Hitachi, Ltd., Tokyo, Japan) [29]. A droplet of nanoparticle dispersion was deposited onto a sample holder and air-dried at 23 °C for 24 h. The morphology of the HA-SH/CS nanoparticles was examined after drying in carbon tape using SEM (EVO 18, Zeiss, Oberkochen, Germany). The composition of the nanoparticles was investigated using a Fourier transform infrared (FT-IR) microscope (iN10, Thermo Fisher Scientific, Inc., Waltham, MA, USA). 

### 2.5. HA-SH/CS Nanoparticle Physical Stability 

The physical stability of the R = 0.10 and R = 0.50 prepared nanoparticles, with or without chemical cross-linking, was assessed under four different conditions: (i) pH = 4.5, (ii) pH = 4.5 and 1× phosphate-buffered saline (PBS), (iii) pH = 7.4, and (iv) pH = 7.4 and 1× PBS. After incubation for 30 min at room temperature, the nanoparticle size, polydispersity index (PDI), and OD_500_ were determined. The OD_500_ of the nanoparticles measured at pH = 4.5 was set as 100%. The extended stability of the HA-SH/CS nanoparticles (pH = 4.5) was also investigated by storing the solution of nanoparticles for three weeks at room temperature. The particle size, PDI, and OD_500_ of the HA-SH/CS nanoparticles were determined according to the OD_500_ measured at 1 d set as 100%.

### 2.6. DOX Loading into HA-SH/CS Nanoparticles

The anthracycline anticancer drug DOX was chosen as a model drug to investigate the drug loading and release characteristics of the HA-SH/CS nanoparticles. The nanoparticles were loaded with 2.0 mg/mL DOX by including the appropriate amount of DOX in the CS solution, which was then mixed with the HA-SH solution and incubated for 24 h at room temperature. The DOX-loaded nanoparticles were separated from the solution by centrifugation at 4500 rpm at 25 °C for 10 min. The supernatant was removed, and the nanoparticles were washed three times with deionized water (pH = 4.5) to remove unbound DOX. The total weight of the empty and drug-loaded nanoparticles was determined after the samples were freeze-dried. The amount of DOX loaded into the HA nanoparticles was calculated by subtracting the mass of DOX in the supernatant from the total mass of DOX in the initial solution, as determined using a UV/VIS spectrophotometer at 480 nm. The percentage of drug-loading content (DLC%) and percentage of drug-loading efficiency (DLE%) were calculated based on the following equations:

(1) $$DLC\%=\frac{Weight\;of\;drug\;in\;nanoparticles\;\left(g\right)}{Total\;weight\;of\;drug\;\left(g\right)-loaded\;nanoparticles\;\left(g\right)}\times 100$$

(2) $$DLE\%=\frac{Weight\;of\;drug\;in\;nanoparticles\;\left(g\right)}{Total\;feed\;weight\;of\;drug\;\left(g\right)}\times 100$$

### 2.7. In Vitro Drug Release from HA-SH/CS Nanoparticles

The release of DOX from HA-SH/CS nanoparticles was investigated in vitro under four different conditions: (i) 1× PBS (pH = 4.5), (ii) 1× PBS containing 10 mM GSH (pH = 4.5), (iii) 1× PBS (pH = 7.4), and (iv) 1× PBS containing 10 mM GSH (pH = 7.4). Briefly, a 3.0 mL release medium was added to 3.0 mg DOX-loaded HA-SH/CS nanoparticles and shaken at 37 °C. At the desired time intervals, the mixture was centrifuged, and 1.0 mL of release medium was removed. The pellets were resuspended in the remaining medium, and the sample was replenished with an equal volume of fresh medium. The concentration of DOX in the collected sample of release medium was determined by UV/VIS spectrophotometry at 480 nm. The release experiments were performed in triplicate.

### 2.8. In Vitro Cytotoxicity 

The cytotoxicity of the empty and DOX-loaded HA-SH/CS nanoparticles was evaluated using 3-(4,5-dimethylthiazol-2-yl)-2, 5-diphenyl tetrazolium bromide (MTT) cell sensitivity assays. Human breast cancer SKBR3 cells were seeded into 96-well cell culture plates at a concentration of 2500 cells/well, and cultured with McCoy’s 5A medium (ATCC) supplemented with 10% FBS and containing R = 0.10 or R = 0.50 DOX-loaded nanoparticles or corresponding empty nanoparticles at different concentrations. Three days later, cell viability was assessed using a CellTiter 96^®^ Aqueous Kit. The optical absorbance at 487 nm was measured using a Varioskan™ Flash microplate reader (Thermo Fisher Scientific, Inc., MA, USA). Non-seeded wells (cell negative) were treated similarly and used as blank controls. The OD_500_ values of the blanks were subtracted from the corresponding samples.

### 2.9. Statistical Analysis 

Statistical analyses were conducted using the SPSS software (IBM, Armonk, NY, USA). Data are presented as the mean ± standard deviation of the values from at least three independent experiments. Differences between the groups were measured using the Student *t*-test. In cases of multi-group testing, one-way analysis of variance and Student –Newman–Keuls post hoc test were conducted. A *p* < 0.05 was considered statistically significant.

## 3. Results and Discussion

### 3.1. Synthesis and Characterization of HA-SH 

**Figure 1:**
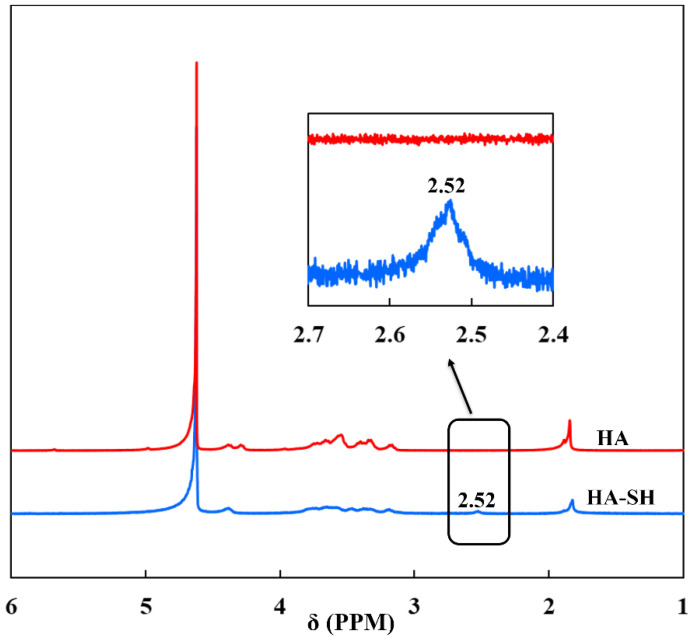
Proton nuclear magnetic resonance (^1^H-NMR) of hyaluronic acid (HA) and thiolated hyaluronic acid (HA-SH), in which δ = 2.52 PPM belongs to the methylene protons of the cysteamine moieties.

Using EDC/HOBt as a coupling agent, HA-SH was synthesized as the carboxylic acid groups of HA reacted with the amine groups of cysteamine dihydrochloride. Disulfide bonds were cleaved to free thiol groups using excess amounts of the reducing reagent DTT. The ^1^H-NMR spectrum of HA-SH is shown in Figure 1. The signal at δ = 2.52 ppm (peak 2) belongs to the methylene protons of the cysteamine moieties. On a molar basis, the degree of the thiol group substitution was 35.1%, and the disulfide moiety was 6.7%, as determined using the Ellman method. 

### 3.2. HA-SH/CS Nanoparticle Fabrication and Characterization 

Dual cross-linked HA-SH/CS nanoparticles were prepared in this study. First, solutions of HA-SH and CS were mixed to generate nanoparticles through electrostatic self-assembly with physical cross-linking between the ionized amino groups of CS (NH_3_^+^) and the ionized carboxyl acid groups (COO^−^) of HA [30,31]. The thiol groups of HA-SH were then oxidized by H_2_O_2_ to form disulfide bonds as chemical cross-linking [32,33]. 

The initial solutions of HA-SH and CS were adjusted to pH = 4.5 to ensure full protonation of the HA-SH and CS. Using solutions with the same pH also avoided any neutralization reactions in the mixing process. 

The fast one-shot addition of one polymer to the other polymer in excess was used instead of dropwise to pass through the flocculation point and generate stable nanoparticles. For the negative nanoparticles, HA-SH was the polymer in excess, while for the positive nanoparticles, CS was the polymer in excess. As a qualitative detection method for the formation of the nanoparticles, optical density was measured at OD_500_ as neither HA-SH nor CS absorb light at this wavelength.

**Figure 2:**
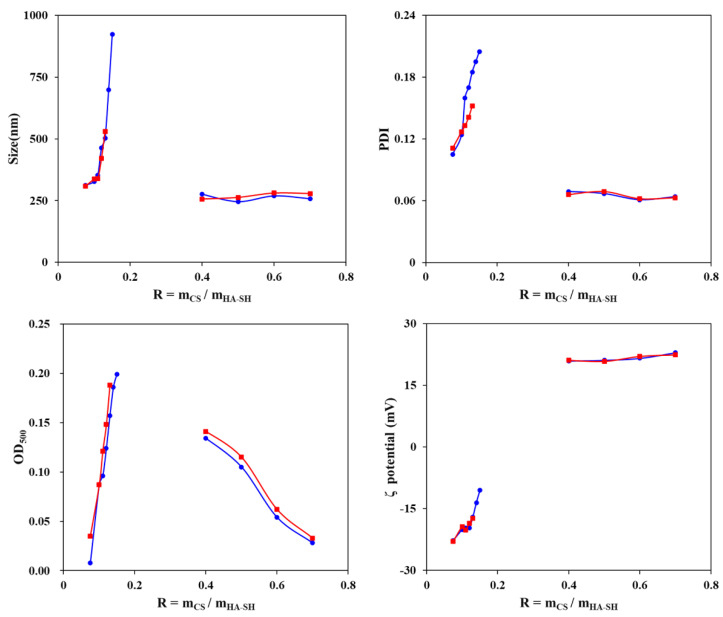
Nanoparticle size, polydispersity index XD(PDI), optical density at 500 nm (OD_500_), and ζ potential of HA-SH/CS nanoparticles as a function of the mass ratio of CS and HA-SH before oxidation (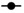) and after oxidation (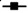).

Nanoparticle formation was studied as a function of the mixing mass ratio m_CS_/m_HA-SH_. The results are reported in particle size, PDI, OD_500_, and ζ potential of dispersions before and after H_2_O_2_ oxidation (Figure 2). Nanoparticles only formed at special R values; thus, the lines of particle size, PDI, OD_500_, and ζ were discontinuous. As the m_CS_/m_HA-SH_ ratio increased, the nanoparticle sizes initially increased, followed by the formation of flocculation and the development of stable dispersions. Surface charge plays an essential role in determining the biological properties of nanoparticles, both in vitro and in vivo [24]. The ζ potential values showed the nanoparticles changed from overall negative charges to positive charges as the m_CS_/m_HA-SH_ ratio increased. H_2_O_2_ oxidation had almost no effect on the particle size, PDI, OD_500_, or ζ potential, indicating the disulfide bonds mainly formed inside the nanoparticles. Furthermore, the Ellman method results showed that all thiols were oxidized to disulfide during the oxidation process. 

**Figure 3:**
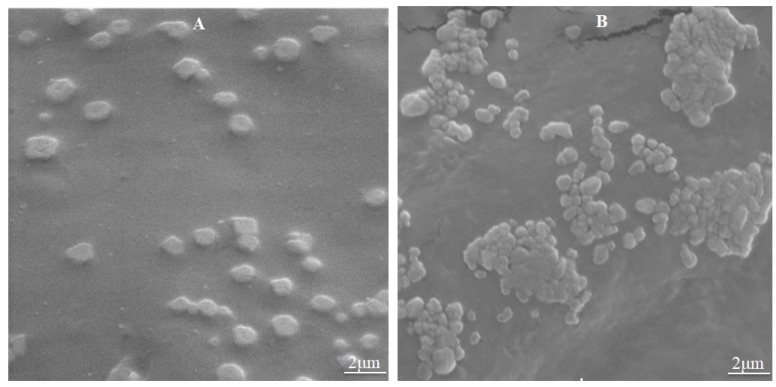
Scanning electron micrographs of HA-SH/CS nanoparticles after drying in carbon tape. (**A**) Negative nanoparticles (R = 0.10). (**B**) Positive nanoparticles (R = 0.50).

Scanning electron micrographs of representative HA-SH/CS nanoparticles are shown in Figure 3. The negative HA-SH/CS nanoparticles (R = 0.10) exhibited a relatively uniform preparation free from aggregates when dried on a surface (Figure 3A). The particles seen in the SEM images were smaller than the range of sizes determined by dynamic light scattering, probably due to the collapse of the nanoparticles upon drying. In contrast, positive HA-SH/CS nanoparticles (R = 0.50) exhibited a mixture of single particles and larger coalesced particles, which consisted of smaller nanoparticles (Figure 3B). 

**Figure 4:**
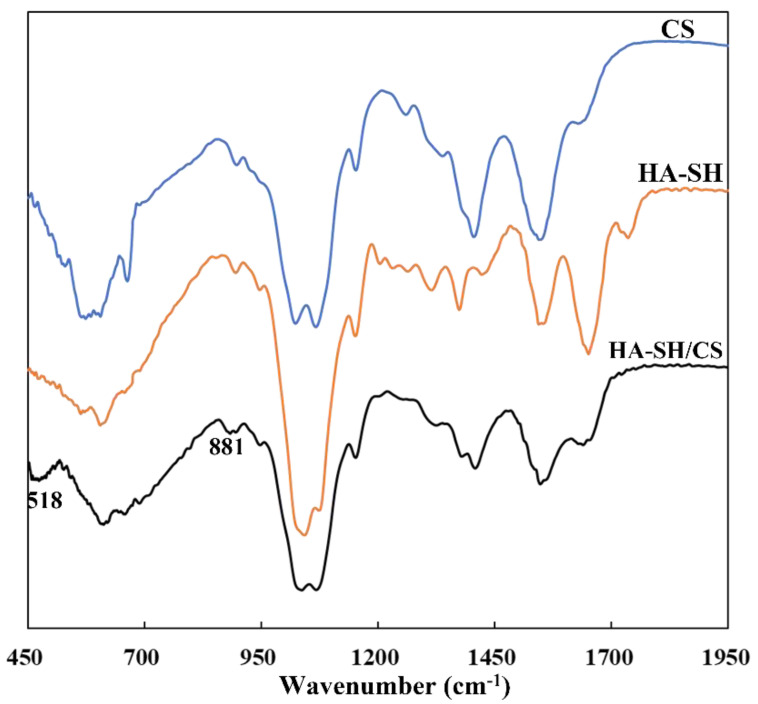
FT-IR spectra of CS, HA-SH, and HA-SH/CS nanoparticle (518 cm^−1^ for disulfide bonds between -SH, and 881 cm^−1^ for Schiff bases between CS-NH_3_^+^ and HA-SH-COO^−^).

A representative FT-IR spectrum of HA-SH/CS nanoparticles is shown in Figure 4. The peak observed at 524 cm^−1^ represents the disulfide bonds formed in the nanoparticles after oxidation. The peak observed at 881 cm^−1^ represents the Schiff bases in the nanoparticles formed between CS-NH_3_^+^ and HA-SH-COO^−^ [34]. 

### 3.3. Nanoparticle Physical Stability 

The formation of polyelectrolyte nanocomplexes is dependent on many factors of the polymer, including the type, MW, and concentration, as well as the process of synthesis, which may vary in the pH of the solution and the mixing ratio [35,36]. In this study, low-molecular-weight CS and ultra-low-molecular-weight HA oligomer (mini-HA) were selected to synthesize self-assembled nanoparticles. 

**Figure 5:**
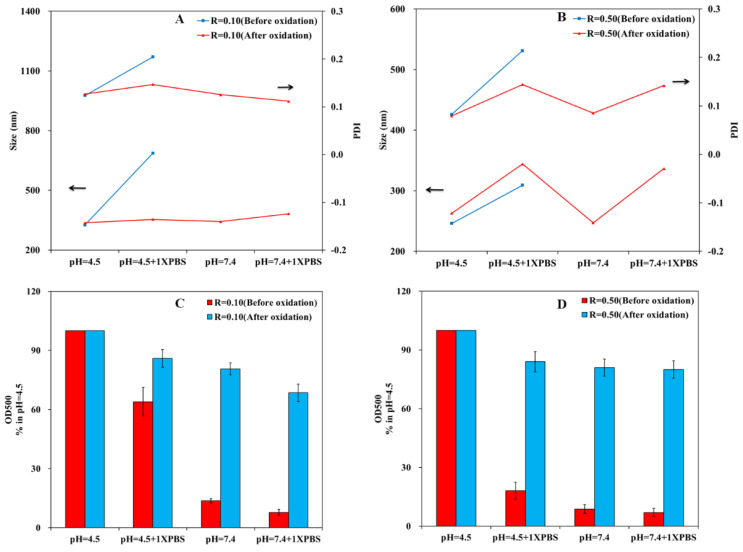
Physical stability of HA-SH/CH nanoparticles under different conditions before and after oxidation. (**A**) and (**C**) Negative nanoparticles (R = 0.10). (**B**) and (**D**) Positive nanoparticles (R = 0.50).

As shown in Figure 5A,C, the OD_500_ of the R = 0.10 and R = 0.50 nanoparticles were unstable at pH = 7.4 and/or 1× PBS without chemical cross-linking. This may be due to the deprotonation of the amine groups in the CS, resulting in the dissociation of the polyelectrolyte complexes. In contrast, particle size, PDI, and OD_500_ of the nanoparticles were minimally affected by pH or ionic strengths after disulfide bond cross-linking (Figure 5B,D). The cross-linking of HA-SH decreased the dissociation process of polyelectrolyte complexes.

**Figure 6:**
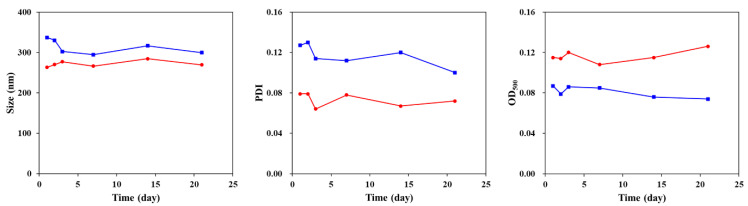
Storage stability (Particle size, PDI, OD_500_) of the HA-SH/CH nanoparticles at pH = 4.5 after oxidation. R = 0.10 (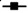) and R = 0.50 (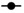).

No apparent alteration in the particle size, PDI, or OD_500_ was observed after three weeks of storage at room temperature following dual cross-linking (Figure 6).

### 3.4. DOX Loading into Nanoparticles

DOX is a weakly basic drug (pKa = 8.2). It was loaded into negative and positive HA-SH/CS nanoparticles at different feed weight ratios at pH = 4.5 (Table 1). The theoretical DLC% = [m_DOX_/((m_CS_ + m_HA-SH_) _in the initial solution_)] ×100. The actual DLC% = [(m_DOX in the initial solution_ - m_DOX in solution after loading_)/((m_CS_ + m_HA-SH_) _in the initial solution_)] ×100. 

For the negative nanoparticles (R = 0.10), the particle size increased, and ζ-potential decreased with increasing DOX DLC%. After loading DOX, the nanoparticles still exhibited negative ζ-potential values, suggesting the negatively charged HA-SH was in excess compared with the levels of the positively charged CS and DOX. Consequently, DOX was both encapsulated inside the nanoparticles and electrostatically bound to excess HA-SH. A DOX DLC% of 20% ultimately led to the formation of aggregates, indicating the negative charge of HA-SH was fully neutralized by DOX under high-DOX loadings, and the nanoparticles were unstable in dispersion.

For the positive nanoparticles (R = 0.50), the particle size and ζ-potential of DOX-loaded nanoparticles remained practically unchanged, with the DOX DLC% ranging from 0% to 10%. In contrast with the R = 0.10 nanoparticles, the R = 0.50 nanoparticles exhibited positive ζ-potential values, suggesting the amount of negatively charged HA-SH was deficient. This also resulted in lower loading efficiencies and reduced levels of loaded content. The amount of DOX incorporated into the particles was strongly affected by both the nanoparticle structure and the feeding ratio relative to the other components in the mixtures.

### 3.5. Drug Release from HA-SH/CS Nanoparticles In Vitro

**Figure 7:**
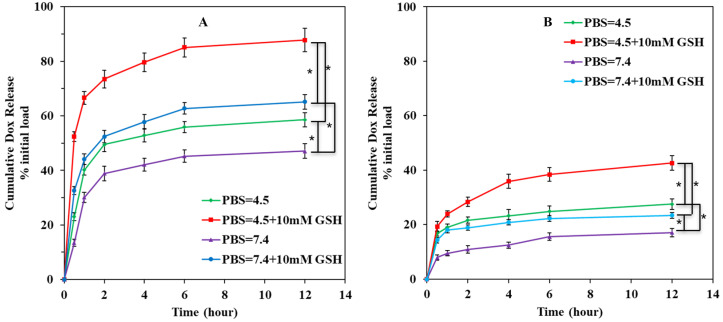
Redox/pH dual-responsive DOX release profiles from DOX-loaded HA-SH/CS nanoparticles under different conditions of pH and GSH concentrations. (**A**) Negative nanoparticle (R = 0.10). (**B**) Positive nanoparticles (R = 0.50). There was a significant difference between different conditions for both nanoparticles (* *p* < 0.05).

Drug release characteristics of the DOX-loaded HA-SH/CS nanoparticles were investigated under different conditions, including various pH and GSH concentrations. The theoretical DLC% was 5%. The accumulative drug release profiles as a function of time are plotted in Figure 7. The release of DOX from the HA-SH/CS nanoparticles was determined to be a redox/pH dual-stimuli-responsive process.

For the negative nanoparticles (R = 0.10), the results showed that under physiological conditions (1× PBS, pH 7.4), approximately 47.1 wt% DOX was released from DOX-loaded nanoparticles over 12 h (Figure 7A). Meanwhile, DOX released from the negative nanoparticles under mildly acidic conditions (1× PBS, pH 4.5) was approximately 58.6 wt% over the same 12 h period, which was significantly higher than the amount of DOX released under the physiological pH (*p* < 0.05). The HA-SH/CS nanoparticles swelled due to protonation of the amine groups in the CS at pH 4.5, which may have promoted DOX release. More importantly, the protonation of the carboxyl groups of HA-SH or deprotonation of the amino groups of DOX under mildly acidic conditions may also have resulted in the faster dissociation of the nanoparticle–DOX complex. In summary, both the swelling and electrostatic repulsion may have contributed to the accelerated release of DOX in the acidic medium compared with that in the physiological medium. 

Similar pH-responsive drug-release profiles were observed for the R = 0.50 positive nanoparticles. However, drug release from the positive nanoparticles was significantly slower than that from the negative nanoparticles (*p* < 0.05). Differences in the location of DOX within the two types of nanoparticles may explain the different release profiles. The DOX in positive nanoparticles was mainly encapsulated inside the hydrophobic core of the nanoparticles, with almost no electrostatic interaction occurring between the nanoparticles and the DOX. In that case, the release kinetics were probably controlled by diffusion.

The drug release characteristics of DOX-loaded HA-SH/CS nanoparticles were also investigated under different GSH concentrations. Intracellular GSH levels in tumor cells are 100–1000-fold higher than that of extracellular levels. This concentration difference can be exploited by using disulfide cross-linked nanocarriers that release their payloads inside cells. As shown in Figure 7A,B, the release of DOX was significantly accelerated in a reducing environment containing 10 mM GSH (*p* < 0.05), which contributed to the cleavage of the disulfide cross-linking bonds. 

Notably, the fastest and most complete drug release was observed at pH 4.5 in the presence of 10 mM GSH, wherein 87.8% of the DOX was released in 12 h (Figure 5A). These results clearly indicated that the redox and pH dual-sensitive degradable nanoparticles in the current study presented a synergistic effect regarding drug release. 

### 3.6. In Vitro Cytotoxicity 

**Figure 8:**
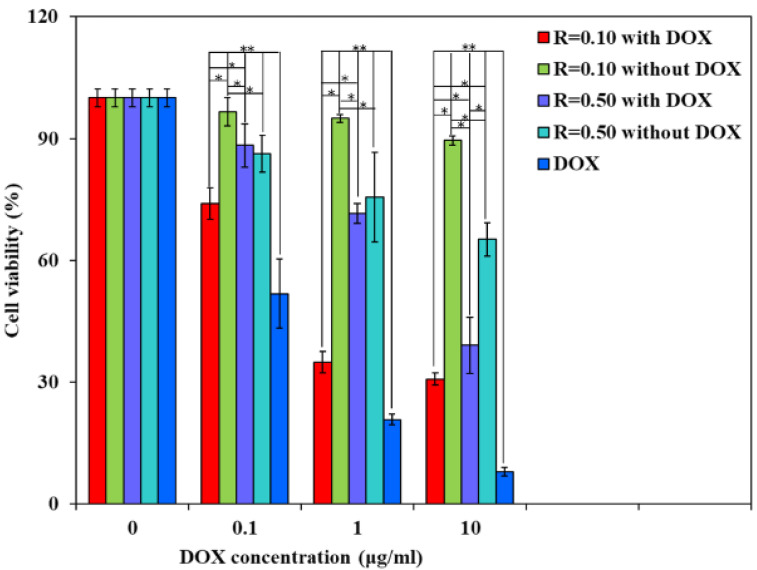
Cell viability of SKBR3 cells after 72 h exposed to DOX-loaded nanoparticles, corresponding empty nanoparticles, or free DOX. There was a significant difference between different nanoparticles with DOX and without DOX (* *p* < 0.05). Furthermore, there was a significant difference between nanoparticles and free DOX (** *p* < 0.05).

To evaluate HA-SH/CS nanoparticles as effective drug carriers for the therapeutic treatment of tumors, in vitro cytotoxicity against the human breast cancer-derived cell line SKBR3 was investigated using MTT assays. As shown in Figure 8, empty R = 0.10 HA-SH/CS nanoparticles, used as a negative control, showed no cytotoxicity, with cell viabilities being ≥90%. However, empty R = 0.50 HA-SH/CS nanoparticles were slightly cytotoxic at the highest concentration, possibly due to the positive charges on the nanoparticle surface [37,38]. A significant inhibition of growth was observed when the SKBR3 cells were treated with either DOX-loaded HA-SH/CS nanoparticles or pure DOX in PBS (pH 7.4), with the inhibition increasing relative to the DOX concentration (*p* < 0.05). These data suggested that the DOX-loaded HA-SH/CS nanoparticles were easily and efficiently taken up by the tumor cells and that they released DOX in an acidic and reduced intracellular microenvironment.

## 4. Conclusions

In this study, new redox/pH dual-responsive nanoparticles with reversible surface charge were prepared using HA-SH and CS for the controlled release of the anticancer drug DOX. The stability of the nanoparticles in physiological media was improved through additional chemical cross-linking of the HA-SH thiol groups. HA-SH/CS nanoparticles possess many favorable traits, such as excellent biocompatibility and biodegradability, CD44 targeting with hyaluronic acid, adequate drug-loading capacity, and rapid drug release in response to intracellular pH and reducing potential. Furthermore, the nanoparticles can be prepared with either negative or positive surface charges according to the requirements of various drugs. These traits provide the HA-SH/CS nanoparticles with characteristics that make them promising candidates for the controlled and targeted delivery of drugs.

## Figures and Tables

**Table 1:** Characteristics of DOX-loaded HA-SH/CS nanoparticles.

Nanoparticles	DLC (wt%)		DLE (%)	Size (nm)	PDI	ζ Potential
	Theory	Actual				
R (m_CS_/m_HA-SH_) = 0.10	0	0	0	337.1	0.127	−19.4
	1	0.61	19.6	341.1	0.114	−18.2
	2	0.99	26.6	362.6	0.129	−18.8
	5	1.71	40.8	423.0	0.123	−15.5
	10	2.08	45.7	582.2	0.144	−14.5
R (m_CS_/m_HA-SH_) = 0.50	0	0	0	263	0.079	20.9
	1	0.38	5.7	263.7	0.078	21.6
	2	0.69	9.76	262.9	0.069	19.8
	5	0.97	13.33	274.2	0.085	21.7
	10	1.07	14.22	272.8	0.107	21.0

## Data Availability

The data presented in this study are available on request from the corresponding author.
